# Greater reduction in the proinsulin-C-peptide ratio with a ketogenic vs control diet in patients with type 2 diabetes

**DOI:** 10.1210/jendso/bvag073

**Published:** 2026-04-21

**Authors:** Marian L Yurchishin, Amanda R Finn, Lauren A Fowler, Sara L Vere-Whiting, Barbara A Gower

**Affiliations:** Department of Nutrition Sciences, University of Alabama at Birmingham, Birmingham, AL 35294, USA; Department of Nutrition Sciences, University of Alabama at Birmingham, Birmingham, AL 35294, USA; Department of Nutrition Sciences, University of Alabama at Birmingham, Birmingham, AL 35294, USA; Department of Nutrition Sciences, University of Alabama at Birmingham, Birmingham, AL 35294, USA; Department of Nutrition Sciences, University of Alabama at Birmingham, Birmingham, AL 35294, USA

**Keywords:** beta-cell, insulin secretion, type 2 diabetes, diet, carbohydrates

## Abstract

**Context:**

The proinsulin to C-peptide (PICP) ratio reflects beta-cell stress and has been shown to decrease following diet-induced weight loss.

**Objective:**

To compare the changes in PICP following a ketogenic (KD) or a low-fat diet (LFD) in individuals with type 2 diabetes (T2D).

**Methods:**

The sample was 55 (±7) years of age, majority female (76%), and 54% African American. Participants (n = 51) were randomized to either a KD or a LFD for 12 weeks. PICP and the acute (ACP) and maximal (CPmax) C-peptide responses were determined using a hyperglycemic clamp. Multiple linear regression analyses were used to examine the effect of diet on changes in PICP. Spearman correlations were conducted to evaluate associations between changes in fasting and post-clamp PICP with changes in beta-cell function.

**Results:**

Diet assignment was a significant predictor of change in fasting PICP (PICP_0_) (KD vs LFD β = −0.18 [−11.16, −0.05]) after adjusting for 12-week fasting glucose and baseline PICP_0_. Results were similar when 190-minute PICP (PICP_190_) was used as the outcome variable (KD vs LFD β = −0.23 [−6.99, −1.19]), after adjusting for 12-week fasting glucose and baseline PICP_190_. Spearman correlation analyses revealed that the change in ACP was inversely associated with changes in both PICP_0_ (ρ = −0.37, *P* = .009) and PICP_190_ (ρ = −0.38, *P* = .01) over the 12-week study.

**Conclusion:**

A KD decreases the proportion of proinsulin secreted to a greater extent than a LFD in patients with early T2D, a change that was associated with an improvement in beta-cell function.

The transition from normal glucose tolerance to overt type 2 diabetes (T2D) necessitates some degree of pancreatic beta-cell failure, leading to persistent hyperglycemia [1-3]. Beta-cell failure is often described as the consequence of “overworked” cells, constantly responding to elevated glucose concentrations until they lose the capacity to adequately secrete insulin [3-5]. However, proper beta-cell function is intricately linked to the insulin sensitivity of other organs, including the liver, where systemic insulin resistance contributes to glucolipotoxicity within the pancreas [3, 5]. Randomized controlled trials have shown that lifestyle interventions prevent or delay T2D development, acting through a combination of multiorgan improvements in insulin sensitivity and reduced insulin demand on the beta-cells [6]. Although numerous lifestyle approaches can benefit patients, evidence indicates that carbohydrate restriction can support weight loss and improve insulin sensitivity in patients with T2D [7]. Accordingly, it is important to examine the spectrum of carbohydrate-restricted diets and their potential to improve beta-cell function.

Carbohydrate-restricted dietary approaches range from moderate reduction (just below 45% of total calories from carbohydrate) to ketogenic diets (KD) that often contain <10% of total calories from carbohydrate and macronutrient replacement primarily from fat (∼60%-70% of total calories) [7]. Moderately carbohydrate-restricted diets vary in their proportions of dietary protein and fat depending on their purpose, such as protein-sparing diets for preoperative bariatric surgery patients or high-fat, moderate protein diets for general weight loss [8]. Low glycemic load diets, including KD, are associated with reduced postprandial glucose concentrations [9], reducing the secretory demand on the beta-cells [10]. KD have also been shown to improve hepatic insulin sensitivity and reduce intrahepatic triglyceride content, potentially leading to lower glucotoxicity and lipotoxicity in the pancreas [11, 12]. Notably, a recent study showed that a KD improved beta-cell function without major weight loss, demonstrating that nutritional ketogenesis may confer benefits beyond its effects on body weight [13]. Given the role of beta-cell function in diabetes progression, further research is needed to clarify how varying levels of carbohydrate intake impact markers of beta-cell health.

Proinsulin is one marker that could reflect clinically meaningful changes in beta-cell stress during diabetes treatment. Proinsulin is the precursor molecule to insulin and is processed into the mature insulin peptide within the beta-cell prior to secretion [14]. As beta-cells lose the ability to effectively process proinsulin into insulin, greater amounts of proinsulin are secreted in children and adults [15-18]. Under eucaloric conditions, moderate carbohydrate restriction appears to reduce proinsulin concentrations to a greater extent compared to standard carbohydrate intake, consistent with the theory that lowering carbohydrate intake reduces beta-cell workload [19]. However, whether KD, with more pronounced reductions in glycemic load and insulin secretion [9, 10], further decrease proinsulin concentrations compared to higher carbohydrate diets remains unknown. Clarifying this relationship could elucidate whether nutritional ketogenesis slows or reverses beta-cell decline in individuals with T2D.

We have previously reported the results of a 12-week intervention that examined the effects of two diets (KD vs low fat diet (LFD)) on ectopic lipid content and beta-cell function in adults with T2D [11, 13]. The objective of this secondary analysis was to compare the changes in fasted and maximal proinsulin responses (proinsulin normalized for C-peptide, PICP) between the diets. We hypothesized that patients randomized to the KD would experience a greater decrease in PICP than those randomized to the LFD. We also explored whether the changes in PICP were associated with changes in measures of beta-cell function from the hyperglycemic clamp.

## Methods

### Study design

The data for this secondary analysis were obtained from a recently completed 12-week randomized clinical trial designed to test the effects of a weight-maintaining KD vs LFD on ectopic lipid content and beta-cell function in adults with T2D (clinical trial registration NCT03430310) [11, 13]. The study was approved by the University of Alabama at Birmingham Institutional Review Board. Written informed consent was obtained from all participants, and the study was conducted in accordance with the Declaration of Helsinki and applicable institutional and federal guidelines.

Study recruitment and data collection took place between October 2018 and July 2023. For the duration of the study, participants received groceries from a local delivery service to prepare their meals and snacks according to their diet assignment. Participants met with a registered dietitian weekly to self-report their weight and return daily food records detailing the foods they ate every day. Based on these visits, a participant's energy prescription was modified to maintain baseline body weight (±1 kg) as much as possible. The KD was compromised of approximately 9% energy from carbohydrates, 65% energy from fat, and 26% energy from protein, and emphasized low-glycemic carbohydrate sources, whole foods, and minimal added sugar. The LFD diet was composed of approximately 55% energy from carbohydrates, 20% energy from fat, and 25% energy from protein, maintaining a similar whole-foods composition, although with a higher proportion of bread, potatoes, and pasta.

### Participants

Participants with T2D managed by diet, metformin, sodium-glucose cotransporter-2 (SGLT-2) inhibitors, dipeptidyl peptidase-4 (DPP-IV) inhibitors, or glucagon-like peptide-1 (GLP-1) receptor agonists were recruited for the parent study. Inclusion criteria were a T2D diagnosis within the past 10 years, hemoglobin A1c (HbA1c) < 8.0% (64 mmol/mol), age 35 to 65 years, BMI 25 to 50 kg/m^2^, self-reported weight stability (<5 kg change in the previous 6 months), and self-reported African-American or European-American race. Exclusion criteria were current glucocorticoid use, tobacco or recreational drug use, and inability to undergo magnetic resonance imaging. Medication use was similar between the diet groups at baseline, including medications for diabetes management and comorbidities. Participants discontinued their medication 1 week prior to baseline testing for metformin and SGLT-2 inhibitors, and 4 weeks prior for DPP-IV inhibitors and GLP-1 receptor agonists.

### Procedures

At baseline and following the 12-week dietary intervention, participants underwent hyperglycemic clamps using a three-stage method described previously [13]. Briefly, the first stage evaluated the first-phase beta-cell response to a glucose challenge. At time 0, a glucose bolus was administered over 2 minutes, followed by an intravenous glucose (D20) infusion to achieve a blood glucose concentration 50 mg/dL (2.78 mmol/L) above fasting for 30 minutes. Blood samples were collected at fasting and then at 2, 4, 6, 8, 10, 15, 20, 25, and 30 minutes after glucose administration. Bolus amounts and glucose infusion rates were estimated using formulas from the RISE consortium [20].

During the second stage, blood glucose was raised to 200 mg/dL (11.10 mmol/L) for 2 hours to estimate insulin sensitivity, with blood sampled every 5 minutes. At minute 150, a second D20 bolus was administered over 2 minutes, and blood glucose was increased to 300 mg/dL (16.65 mmol/L) for the third stage. Blood was sampled every 5 minutes from minutes 150-180. After 30 minutes at 300 mg/dL, 5-g arginine (10% saline) was infused to evaluate maximal beta-cell response, where blood was sampled every 2 minutes from minutes 182-190.

### Serum assays

Serum samples were stored at −70 °C until analysis. Glucose was measured using a SIRRUS chemistry analyzer (Stanbio Laboratory) with glucose oxidase reagent (minimum detectable value: 2 mg/dL; interassay CV: 4.48%; intra-assay CV: 1.28%). Proinsulin was measured in duplicate using Millipore Human Proinsulin RIA kits (Billerica, MA) (minimum sensitivity: 6.0 pM; intra-assay CV: 3.56%; inter-assay CV: 5.13%) (catalog No. HPI-15K, RRID: AB_2891152). C-peptide was analyzed on the TOSOH platform (minimum sensitivity: 0.04 ng/mL; interassay CV: 6.81%; intra-assay CV: 1.67%) (catalog No. 25284, RRID: AB_3107182).

### Calculations

Calculations for acute/first-phase C-peptide response (ACP) and maximal C-peptide response (CPmax) were derived from the RISE protocol [20]. ACP was calculated as the mean incremental response above baseline during minutes 2-10, and CPmax as the mean incremental response during minutes 184-190 above the mean at minutes 170-180. PICP ratios were calculated as the molar ratio of proinsulin to C-peptide at minutes 0 and 190 minutes obtained from the clamp. The PICP ratio was demonstrated to be a stronger predictor of incident T2D compared to proinsulin-to-insulin [15], supporting the relevance of PICP as a marker for future beta-cell deficiency.

### Statistical analysis

Diet group differences in descriptive characteristics were assessed with independent sample *t*-tests for continuous variables and Pearson's chi-square test for categorical variables. Multiple linear regression models were used to test whether the diet group was associated with changes in PICP_0_ and PICP_190_. Covariates were 12-week fasting glucose and baseline PICP values. The inclusion of 12-week fasting glucose as a covariate was due to differences in fasting glucose levels between diet groups at weeks 0 and 12, as it has been reported that elevated proinsulin levels are directly associated with hyperglycemia [21]. Weight change was considered as a covariate, but it was not correlated with changes in PICP outcomes; hence, it was removed from regression models to increase statistical power. Assumptions for normality of continuous variables, residual normality, and homoscedasticity were confirmed for all models using the Shapiro-Wilk test, residual histograms, and scale-location plots, respectively. All models were assessed for multicollinearity and yielded a variance inflation factor of <5. Spearman correlations were used to assess associations between baseline PICP (PICP_0_, PICP_190_) and baseline beta-cell function (ACP, CPmax), as well as the associations between change scores (week 12: baseline) for those variables. Significance was set at *P* < .05 for all statistical tests. All analyses were performed with R version 4.5.2 (R Core Team, 2025). Figures were generated using Sigmaplot v16.0.

## Results

**Figure 1:**
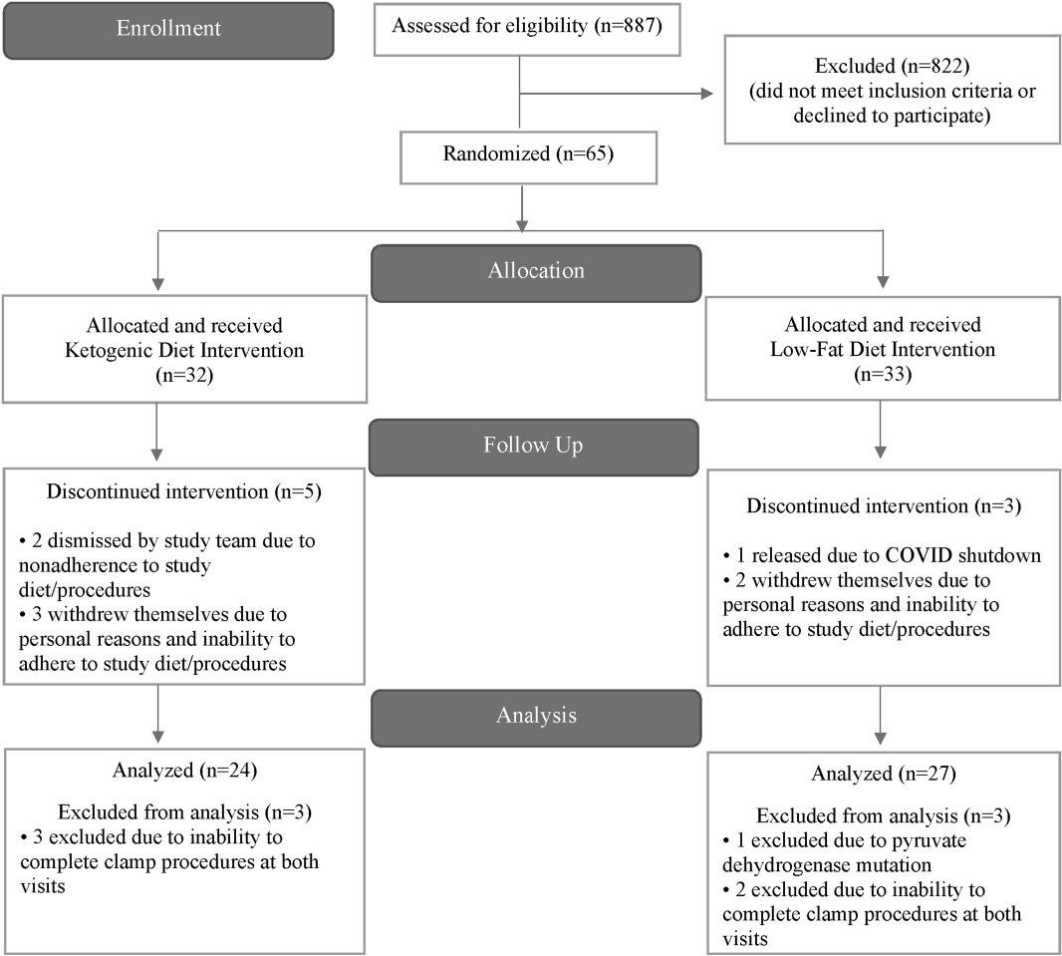
Consolidated standards of reporting trials (CONSORT) diagram.

Sixty-five participants were enrolled in the parent study. Five participants withdrew due to inability to adhere to study protocol and personal reasons, two participants were dismissed due to nonadherence to study protocol, and one was released due to COVID shutdown. Of the 57 participants who completed the 12-week diet intervention, one participant was excluded due to having a pyruvate dehydrogenase mutation, and 5 were unable to complete the clamp procedure at both visits due to loss of venous access. The final number of participants included in this analysis was 51 (See Consolidated Standards of Reporting Trials [CONSORT] diagram [Fig. 1]).

Descriptive characteristics of the overall cohort and diet groups are presented in Table 1. Participants within the LFD had lower fasting glucose levels at both baseline and 12 weeks, but the change in glucose values over the intervention did not differ by group. Baseline PICP_0_ and PICP_190_ were slightly lower in the LFD group, but these differences were not statistically significant between groups.

**Table 1:** Descriptive statistics of the overall sample and by diet assignment

	Overall (N = 51)	Ketogenic Diet (N = 24)	Low-Fat Diet (N = 27)
Age, yrs	54.5 ± 6.9	53.7 ± 6.2	55.3 ± 7.5
Sex M/F	10/41	4/20	6/21
Race, AA/EA	18/33	10/14	8/19
Baseline weight, kg	98.9 ± 17.7	98.6 ± 19.8	99.2 ± 16.0
12-week weight, kg	94.5 ± 17.1	95.1 ± 19.2	94.0 ± 15.4
Baseline BMI, kg/m^2^	35.8 ± 6.6	35.4 ± 6.6	36.0 ± 6.7
12-week BMI, kg/m^2^	34.3 ± 6.3	34.1 ± 6.4	34.6 ± 6.3
Baseline fasting glucose, mg/dL	150.7 ± 36.7	163.0 ± 41.5	139.8 ± 28.3*
12-week fasting glucose, mg/dL	131.3 ± 30.3	142.1 ± 37.2	121.8 ± 18.4*
Baseline PICP_0_	36.32 ± 19.79	41.0 ± 23.1	32.1 ± 15.5
12-week PICP_0_	26.41 ± 12.43	26.7 ± 13.6	26.1 ± 11.6
Baseline PICP_190_	23.20 ± 12.54	26.0 ± 12.2	20.7 ± 12.5
12-week PICP_190_	17.87 ± 7.70	18.3 ± 8.5	17.5 ± 7.1
Baseline ACP, ng/mL	0.27 ± 0.76	0.20 ± 0.91	0.33 ± 0.62
12-week ACP, ng/mL	0.39 ± 0.53	0.31 ± 0.38	0.46 ± 0.63
Baseline CPmax, ng/mL	7.16 ± 4.22	6.07 ± 3.75	8.12 ± 4.44
12-week CPmax, ng/mL	7.92 ± 3.78	7.19 ± 2.59	8.54 ± 4.51

Data are presented as mean ± SD unless otherwise noted.

Abbreviations: AA, African American; ACP, Acute C-peptide response; BMI, body mass index; CPmax, maximal C-peptide response; EA, European American; F, female; PICP, proinsulin to C-peptide ratio.

^*^ *P* < .05 for between-group difference.

**Figure 2:**
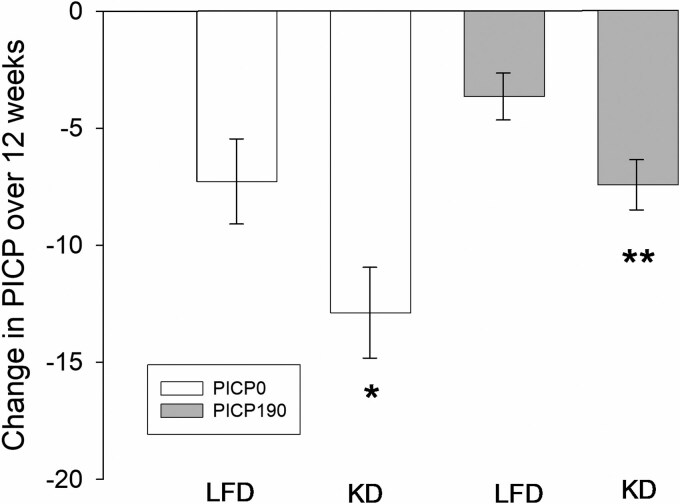
Estimated marginal means for change in PICP_0_ and PICP_190_, stratified by diet group. Means are adjusted for baseline values and 12-week fasting glucose. *, **P < .05, .01, respectively, for effect of diet. Abbreviations: KD, ketogenic diet; LFD, low-fat diet.

Results from the multiple regression analyses are displayed in Table 2 and Fig. 2. Diet assignment significantly predicted change in PICP_0_ after adjusting for 12-week fasting glucose and baseline PICP_0_ (−0.18; 95% CI, −11.16 to −0.05). Participants randomized to the KD experienced a 56% greater decrease in PICP_0_ compared with those randomized to the LFD (*P* < .05). The results were similar when PICP_190_ was the outcome variable. After adjusting for 12-week fasting glucose and baseline PICP_190_, the diet group was correlated with the change in PICP_190_ (−0.23; 95% CI, −6.99 to −1.19), and there was a 49% greater decrease in PICP_190_ in the KD compared with the LFD group (*P* < .01).

**Table 2:** Multiple linear regression models predicting changes in PICP_0_ and PICP_190_

Outcome	Predictor	β	95% CI	*P*
Change in PICP_0_	R^2^ = 0.82			
	Diet			
	Low-Fat	Reference		
	Ketogenic	−0.18	(−11.16, −0.05)	.048
	12-Week Fasting glucose	0.25	(0.04, 0.22)	.007
	Baseline PICP_0_	−0.77	(−0.73, −0.46)	<.001
Change in PICP_190_	R^2^ = 0.87			
	Diet			
	Low-Fat	Reference		
	Ketogenic	−0.23	(−6.99, −1.19)	.007
	12-Week Fasting glucose	0.36	(0.06, 0.16)	<.01
	Baseline PICP_190_	−0.87	(−0.75, −0.52)	<.001

Spearman correlation analyses (Table 3) revealed that baseline PICP_0_ was inversely associated with CPmax (*P* < .05), whereas PICP_190_ was inversely associated with both ACP and CPmax (*P* < .001 for both). Change in PICP_0_ was inversely correlated with change in ACP, indicating that participants with greater decreases in fasting PICP over the intervention had larger increases in the first-phase C-peptide response (*P* < .01). However, changes in PICP_0_ were not significantly associated with change in the maximal C-peptide response to glucose (*P* = .05). In contrast, change in PICP_190_ was inversely correlated with change in both ACP (*P* = .01) and CPmax (*P* < .001), indicating that decreases in post-challenge PICP were associated with increases in both beta-cell function measures.

**Table 3:** Spearman correlation coefficients for the associations among PICP measures and beta-cell function measures among all participants combined

Correlation	Baseline Values	Change Scores
PICP_0_ × ACP	−0.19	−0.37**
PICP_190_ × ACP	−0.46*	−0.38*
PICP_0_ × CPmax	−0.28*	−0.28
PICP_190_ × CPmax	−0.53***	−0.48***

^*^, **, ***P < .05, .01, .001, respectively.

## Discussion

This secondary analysis was conducted to determine whether a KD would elicit greater decreases in PICP compared to the LFD in patients with T2D. The results indicated that exposure to the KD produced a 56% greater decrease in the proportion of proinsulin secreted compared to the LFD. Furthermore, the decrease in PICP was associated with improvements in hyperglycemic clamp measures of acute and maximal C-peptide responses to glucose. These results suggest that the KD led to significant reductions in beta-cell stress as reflected in PICP, and therefore, it may be a therapeutic option for individuals with T2D to minimize disease progression. Subsequent studies are needed to compare a KD against moderately carbohydrate-restricted diets to determine if reductions in PICP parallel reductions in dietary carbohydrate.

Previous studies have demonstrated the value of assessing proinsulin concentrations in the context of changing disease status [15], but a few have examined proinsulin or PICP during diet interventions. In Denmark, researchers examined PICP in patients with T2D after 2 6-week randomized controlled diet interventions. Patients were assigned to either a reduced carbohydrate, high protein diet (30% carbohydrate: 30% protein: 40% fat) or a conventional diet (50% carbohydrate: 17% protein: 33% fat) under either eucaloric or hypocaloric conditions. In the eucaloric study, beta-cell responsiveness was greater, and PICP was lower following the reduced carbohydrate diet compared with the conventional diet [19]. In contrast, during the hypocaloric intervention, both diet groups had significant reductions in their PICP, but no differences were detected between groups in PICP reduction or the beta-cell response to glucose [22]. The correlation between diet-induced weight loss and reduced proinsulin has been established in other studies focused on diet therapy for patients with newly diagnosed T2D [23, 24]. Thus, it is possible that under hypocaloric conditions, the effects of weight loss on beta-cell stress overshadowed the potential effects of carbohydrate restriction. However, in contexts where clinically meaningful weight loss is not possible, the carbohydrate quantity and glycemic load of the diet may play important roles in alleviating beta-cell stress.

The present study also documented that a change in PICP_0_ was associated with a change in ACP, whereas a change in PICP_190_ was associated with changes in both ACP and CPmax. There was greater variability in the PICP_0_ measure compared to the PICP_190_ measure, likely due to the tighter control of glucose during the clamp compared to the fasted state. This greater variability may have affected the ability to detect associations, perhaps explaining the lack of association of PICP_0_ with CPmax. Alternatively, these observations may imply that fasted proinsulin processing is more related to first-phase insulin secretion, whereas post-challenge PICP may more broadly reflect beta-cell function across the continuum from fasted to fed. A sufficient first phase insulin response is critical for managing glycemia [25, 26]; however, first-phase secretion is not simple to measure outside of clinical research settings. In this regard, fasted proinsulin and C-peptide could readily be measured in sera from a clinic-based blood collection and be used to gauge lifestyle intervention effectiveness. We measured PICP at 12 weeks following the start of the diet intervention; thus, we cannot report on the time course of change in PICP. Further research is needed to determine the optimal time period for assessing PICP following intervention initiation.

The present study has strengths and limitations. Strengths include the use of a multiphase hyperglycemic clamp to measure beta-cell function, that the diets were provided to participants for the duration of the study, and the inclusion of African- and European-American participants. The hyperglycemic clamp provides a uniform glucose stimulus across all study participants, allowing for first-phase and maximal insulin secretion to be evaluated and compared without confounding by individual variability in ambient glucose concentration. Limitations include the low number of male participants and limited generalizability to individuals with more advanced disease, where beta-cell health could be more difficult to improve with diet alone.

Additionally, although decreased PICP was associated with improved beta-cell function, it is possible that decreased PICP primarily represents reduced beta-cell oxidative stress. By reducing oxidative stress, the KD may reduce an upstream insult that ultimately impacts insulin secretion. Lifestyle interventions may reduce glucolipotoxicity prior to clinically meaningful improvements in insulin secretion capabilities, and it is unclear whether PICP improvements occurred prior to or alongside the beta-cell function improvements. Further research is needed to determine whether changes in PICP are mechanistically related to beta-cell function.

In conclusion, a KD reduced PICP to a greater extent compared to a LFD in patients with T2D. Furthermore, changes in PICP were significantly associated with changes in beta-cell function during the hyperglycemic clamp, showing that a decrease in PICP was correlated with an increase in beta-cell function. Taken together, these observations suggest that a KD may reduce beta-cell stress and that PICP could be a clinically accessible means of assessing changes in beta-cell function in patients with T2D.

## Data Availability

Some or all datasets generated during and/or analyzed during the current study are not publicly available but are available from the corresponding author on reasonable request.

## Clinical Trial Registration

Clinical Trial Registration number: NCT03430310.

## Abbreviations

KD: ketogenic diet
LFD: low-fat diet
PICP: proinsulin-to-C-peptide
T2D: type 2 diabetes
